# First comprehensive analysis of lysine acetylation in *Alvinocaris longirostris* from the deep-sea hydrothermal vents

**DOI:** 10.1186/s12864-018-4745-3

**Published:** 2018-05-10

**Authors:** Min Hui, Jiao Cheng, Zhongli Sha

**Affiliations:** 1 Laboratory of Marine Organism Taxonomy and Phylogeny, Institute of Oceanology, Chinese Academy of Sciences, Qingdao, 266071 China; 2 University of Chinese Academy of Sciences, Beijing, 100049 China; 3 Center of Deep Sea Research, Institute of Oceanology, Chinese Academy of Sciences, 7 Nanhai Road, Qingdao, 266071 China

**Keywords:** Alvinocarididae, Hemocyanins, Hydrothermal vent, Lysine acetylation, Post-translational modification

## Abstract

**Background:**

Deep-sea hydrothermal vents are unique chemoautotrophic ecosystems with harsh conditions. *Alvinocaris longirostris* is one of the dominant crustacean species inhabiting in these extreme environments. It is significant to clarify mechanisms in their adaptation to the vents. Lysine acetylation has been known to play critical roles in the regulation of many cellular processes. However, its function in *A. longirostris* and even marine invertebrates remains elusive. Our study is the first, to our knowledge, to comprehensively investigate lysine acetylome in *A. longirostris*.

**Results:**

In total, 501 unique acetylation sites from 206 proteins were identified by combination of affinity enrichment and high-sensitive-massspectrometer. It was revealed that Arg, His and Lys occurred most frequently at the + 1 position downstream of the acetylation sites, which were all alkaline amino acids and positively charged. Functional analysis revealed that the protein acetylation was involved in diverse cellular processes, such as biosynthesis of amino acids, citrate cycle, fatty acid degradation and oxidative phosphorylation. Acetylated proteins were found enriched in mitochondrion and peroxisome, and many stress response related proteins were also discovered to be acetylated, like arginine kinases, heat shock protein 70, and hemocyanins. In the two hemocyanins, nine acetylation sites were identified, among which one acetylation site was unique in *A. longirostris* when compared with other shallow water shrimps. Further studies are warranted to verify its function.

**Conclusion:**

The lysine acetylome of *A. longirostris* is investigated for the first time and brings new insights into the regulation function of the lysine acetylation. The results supply abundant resources for exploring the functions of acetylation in *A. longirostris* and other shrimps.

**Electronic supplementary material:**

The online version of this article (10.1186/s12864-018-4745-3) contains supplementary material, which is available to authorized users.

## Background

Deep-sea hydrothermal vent is one of the chemosynthetically-driven ecosystems and characterized with high pressure, continuous darkness, and enriched hydrogen sulfide (H_2_S), methane (CH_4_), heavy metals in the ejected fluid [1]. Surprisingly, particular animals are thriving under these harsh conditions, which must develop specific mechanisms to tolerate the extremes and yet benefit from the chemoautotrophic production. As the development of sequencing technology and bioinformatics, large genomic resources have been discovered in hydrothermal vent faunas, such as mussels [2–4], worms [5], crabs [6] and shrimps [7], which have greatly expanded our understanding for the molecular mechanisms of their adaptation to these extreme environments. However, proteomics researches in hydrothermal vent animals have received relatively little attention.

Post-translational modifications (PTMs) modulate the activity of proteins in most eukaryotes [8, 9]. Lysine acetylation is a dynamic and reversible PTM involved in a broad array of biological functions, e.g. gene regulation, DNA-protein interactions, subcellular localization, and protein stability [10]. Changes in cellular lysine acetylation status can also alter metabolic enzyme activity and provide a metabolic adaptive mechanism for the cells [11–14]. Besides, more studies indicate that lysine acetylation plays pivotal roles in mitochondrial function and stress response [15–17]. Despite intensive research on lysine acetylation over the past decade, most studies for faunas have been performed in mammalians. Recently, with the combination of antibody-based affinity enrichment and high sensitive mass spectrum (MS), high-throughput lysine acetylome analyses are available and becoming popular [18–21]. However, there is no comprehensive study for any marine animal species.

Shrimps of the family Alvinocarididae are specifically inhabiting in deep-sea hydrothermal vent and cold seep chemosynthetic areas [22]. Among them, *A. longirostris* is the only species found co-distributed in both environments thus far [23, 24], and is mostly found on the mussel beds surrounding the base of active hydrothermal chimneys, suggesting they can survive in a fairly reducing environment [25]. These characters make them an excellent model for studying extreme environmental adaptation mechanisms. In this study, we globally identified lysine acetylation sites in proteins of *A. longirostris* from the Iheya North hydrothermal vent in Okinawa Trough by taking advantage of affinity enrichment and LC (liquid chromatography)-MS/MS, and characterized the protein functions systematically. Further study on the role of lysine acetylation in the shrimp can enhance our understanding of how marcoorganisms have adapted to deep-sea extreme environments.

## Methods

### Sample collection and protein extraction

**Fig. 1:**
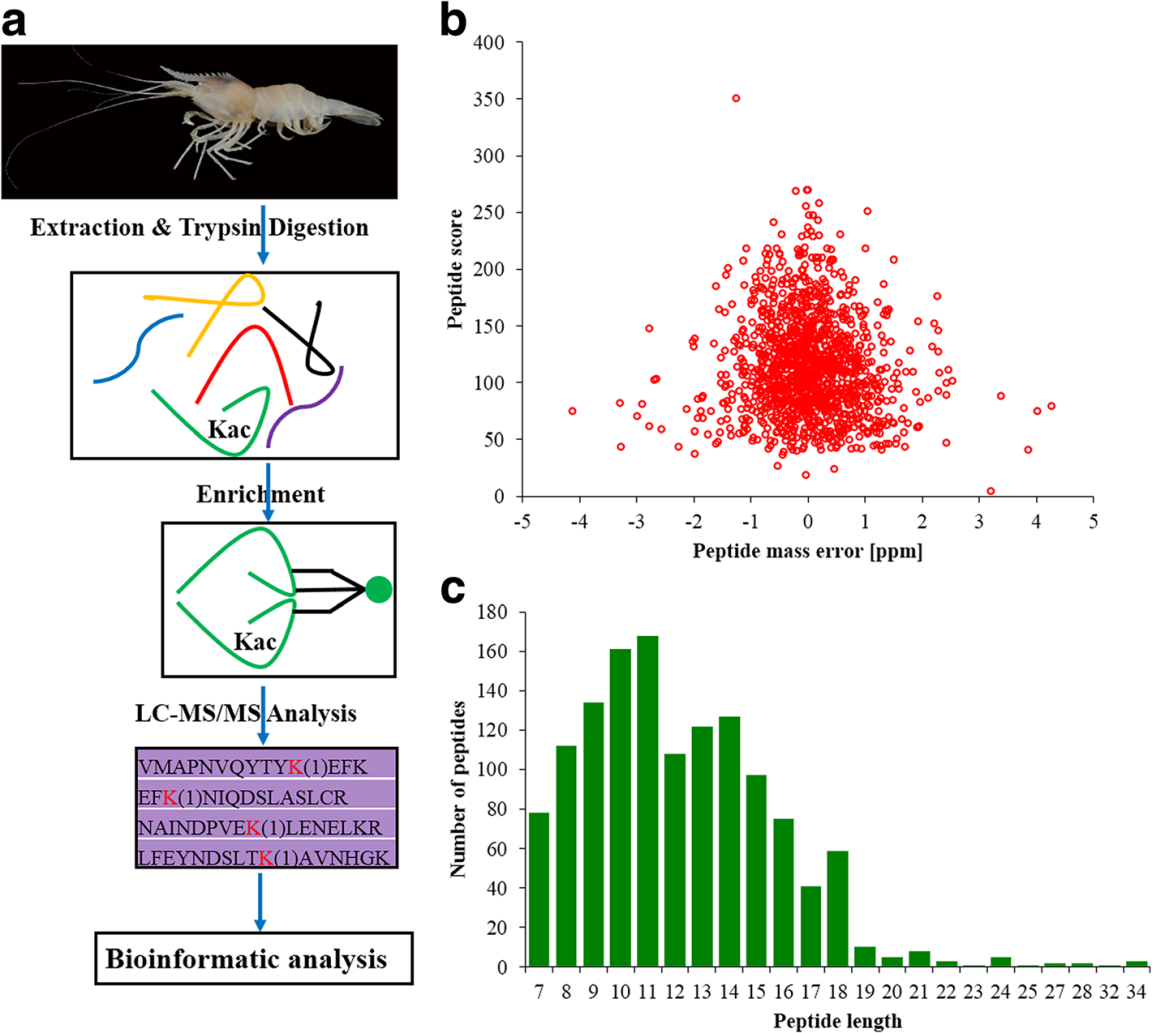
Overview of the global identification of lysine acetylation sites in *Alvinocaris longirostris*. **a** General workflow for the acetylome analysis. **b** Mass error distribution of the identified peptides. **c** Length distribution of the peptides

Samples of *A. longirostris* were collected in August, 2016 during the cruise by the scientific research vessel (RV) KEXUE (Institute of Oceanology, Chinese academy of Sciences, China). The sampling sites were near the Iheya North hydrothermal vent in Okinawa Trough (27°16.2′ N, 127°04.6′ E, depth 1329 m). Materials were taken by the remotely operated vehicle (ROV) Quasar MkII of SMD in the United Kingdom, which was deployed using the RV KEXUE. The in situ environmental conditions were detected by using the sensors of the ROV. The temperature was ~ 3.82 °C, the conductivity was ~ 5.85 s/m, the dissolved carbon dioxide was ~ 304 ppm and the methane concentration was ~ 3.92 mmol/l, while many other data was missing. The samples were immediately frozen in liquid nitrogen and stored at − 80 °C. After the cruise, the abdomens of shrimps were dissected for protein extraction in the lab. The overall technological process was outlined in Fig. 1a.

Protein of two shrimps was extracted separately as two biological replicates. The tissue was grinded into powder with liquid nitrogen. Four volumes of lysis buffer (8 M urea, 1% Triton-100, 10 mM dithiothreitol, and 1% Protease Inhibitor Cocktail) with inhibitors (3 μM TSA and 50 mM NAM) was added to the powder, followed by sonication three times on ice. The residues were removed by centrifugation at 20,000 g, 4 °C for 10 min. The protein was then precipitated with cold 20% TCA for 2 h at − 20 °C. After centrifugation at 12,000 g, 4 °C for 10 min, the supernatant was discarded. The remaining precipitate was washed with cold acetone for three times, and the protein was finally re-dissolved in 8 M urea. The protein concentration was measured with BCA kit accordingly.

### Trypsin digestion and HPLC fractionation

Before digestion, the protein solution containing approximate 10 mg protein was reduced with 5 mM dithiothreitol for 30 min at 56 °C and then alkylated with 11 mM iodoacetamide for 15 min at room temperature in darkness. The urea concentration of the protein solution was then diluted to less than 1 M by adding 100 mM NH_4_HCO_3_. Trypsin (V5111, Promega, Madison, USA) was first added at 1:50 trypsin-to-protein mass ratio and the sample was digested overnight. For the second time, 1:100 trypsin-to-protein mass ratio was selected and the digestion lasted for 4 h. The tryptic peptides were then fractionated into fractions by high pH reverse-phase HPLC using Thermo Betasil C18 column (5 μm particles, 10 mm internal diameter, 250 mm length). Peptides were first separated into 60 fractions with a gradient of 8 to 32% acetonitrile (pH 9.0) for 60 min. After that, the peptides were combined into four fractions and dried by vacuum centrifuging.

### Affinity enrichment for acetylated peptides

The peptides were dissolved in NETN buffer (100 mM NaCl, 1 mM EDTA, 50 mM Tris-HCl, 0.5% NP-40, pH 8.0) and incubated with pre-washed antibody beads (Lot number PTM104, PTM Bio, Hangzhou, China) at 4 °C overnight. During the incubation, it was shaken gently. The beads were washed four times with NETN buffer and twice with ddH_2_O. The bound peptides were eluted with 0.1% trifluoroacetic acid. Then they were combined and vacuum-dried. The enriched peptides were desalted with C18 ZipTips (Millipore) accordingly and submitted to LC-MS/MS analysis.

### LC-MS/MS analysis

The peptides were dissolved in 0.1% formic acid (solvent A) and separated on an EASY-nLC 1000 UPLC system. The gradient of solvent B (0.1% formic acid in 98% acetonitrile) included an increase from 6 to 23% over 26 min, 23 to 35% in 8 min, climbing to 80% in 3 min, holding at 80% for the last 3 min, and finally at a constant flow rate of 400 nl/min.

The peptides were subjected to nanospray ionization (NSI) source followed by tandem mass spectrometry (MS/MS) in Orbitrap Fusion™ Tribrid™ (Thermo). The electrospray voltage was set as 2.0 kv. For MS scans, the scan range was 350 to 1550 m/z. Intact peptides were detected with a resolution of 60,000 in the Orbitrap. Peptides were selected for MS/MS with normalized collisional energy (NCE) being set as 35 and ion fragments were detected with a resolution of 15,000. A data-dependent procedure that alternated between one MS scan and the following 20 MS/MS scans was processed for the top 20 precursor ions above a threshold intensity of 5E3 in the MS scan with 15.0 s dynamic exclusion. Automatic gain control (AGC) was performed to prevent overfilling of the Orbitrap and 5E4 ions were accumulated for generation of MS/MS spectra.

### Database search and motif analysis

The identified MS/MS data were analyzed by Maxquant search engine (v.1.5.2.8) [26, 27]. Tandem mass spectra were searched against a protein database of *A. longirostris* concatenated with reverse decoy database. The database was constructed with the deduced peptide sequences (45,313) from the transcriptome of *A. longirostris* created by our lab (accession number SRX3177689, unpublished). Trypsin/P was specified as cleavage enzyme allowing up to four missing cleavages. The mass tolerance was set as 20 ppm in first search and 5 ppm in main search for precursor ions, and 0.02 Da for fragment ions. Carbamidomethyl on Cys was specified as fixed modification, while oxidation on Met, acetylation on Lys and acetylation on protein N-terminal were specified as variable modifications. False discovery rate (FDR) was adjusted to <1% and minimum score for modified peptides was set >40. The models of protein sequences constituted with amino acids in specific positions of modify-21-mers (10 amino acids upstream and downstream of the site) were analyzed by using motif-x (http://motif-x.med.harvard.edu/motif-x.html). In order to further investigate the lysine acetylation profile of the microbial community of *A. longirostris*, tandem mass spectra were similarly searched against the protein database of the family Methylococcaceae downloaded from Uniprot, considering the high concentration of methane (~ 3.92 mmol/l) in the sampling sites.

### Protein annotation and functional enrichment

Different databases were selected for protein functional annotation. Gene Ontology (GO) annotation proteome was derived from the UniProt-GOA database (http://www.ebi.ac.uk/GOA/). If the proteins were not annotated by UniProt-GOA database, the InterProScan (https://www.ebi.ac.uk/interpro) would be applied to annotate by protein sequence alignment method, which was also used for protein domain functional description. Then proteins were then classified by GO annotation (http://www.geneontology.org/). For each GO and protein domain category, a two-tailed Fisher’s exact test was employed to test the enrichment of the acetylated proteins against all predicted proteins from the transcriptome of *A. longirostris*. The item with a corrected *p*-value < 0.05 was considered significant. Further, KEGG (Kyoto Encyclopedia of Genes and Genomes; http://www.genome.jp/kegg/) online service tools KAAS was utilized to predict the pathways in which the acetylated proteins were involved. Then the annotation results were mapped on the KEGG pathway database using KEGG mapper. These pathways were also enriched as performed in GO enrichment and further classified into hierarchical categories according to the KEGG website. Subcellular localizations of the protein were predicted by Wolf PSORT (http://wolfpsort.seq.cbrc.jp/) [28].

### Protein-protein interaction (PPI) network of acetylated proteins

Due to absence of protein information of *A. longirostris* in the STRING database, all identified acetylated protein sequences in *A. longirostris* were aligned with protein data of its relatively closely related species, *Daphnia pulex*. The matched homologous proteins in *D. pulex* were then searched against the STRING database version 10.5 for protein-protein interaction analysis. Only interactions between the proteins belonging to the searched data set were selected, and thereby external candidates were excluded. Interactions that had a confidence score ≥ 0.4 (high confidence) were extracted. Interaction network form STRING was visualized in Cytoscape [29]. A graph theoretical clustering algorithm, molecular complex detection (MCODE) in Cytoscape was applied to analyze densely connected regions.

### Structure and evolution analyses of hemocyanin

According to annotation, deuced amino acid sequences of two hemocyanins with acetylation sites were identified, which were described as hemocyanin (AlHc) and hemocyanin subunit 1 (AlHc1). Secondary structures of the two proteins were predicted by NetSurfP [30]. The presumed tertiary structure was established using SWISS-MODEL prediction algorithm (http://swissmodel.expasy.org/) and displayed by DeepView/Swiss-Pdb Viewer version 4.0.2. The two hemocyanin sequences were aligned with those downloaded from GenBank of other caridea shrimps using MEGA version 6.0 [31]. Phylogenetic trees were constructed using Maximum Likelihood (ML) method with RAxML Black-Box webserver (http://phylobench.vital-it.ch/raxml-bb/) [32]. For the ML analysis, we selected the Gamma model of rate heterogeneity and assessed support for the tree nodes using bootstrap procedure with 1000 heuristic replicates.

## Results and discussion

### The first lysine acetylome map of the deep-sea hydrothermal vent shrimp

Deep-sea hydrothermal vents are extreme chemosynthetic ecosystems where abundant endemic animals inhabit, including many crustaceans, like shrimps. However, lack of genomic information impedes the clarification of molecular mechanisms in the adaptation to these harsh environments. As one way of the PTMs, protein acetylation has been revealed to play critical roles in various physiological processes related to adaptive reaction [33–35]. Recently, we have sequenced the transcriptome of *A. longirostris* and identified the protein coding domains, which supply baseline data for PTM studies. Therefore, we characterized the lysine acetylated proteins in *A. longirostris* systematically by utilizing highly sensitive immuno-affinity purification in combination with high-resolution LC-MS/MS in order to facilitate further research for their biological functions in the adaptation of the hydrothermal shrimps.

**Fig. 2:**
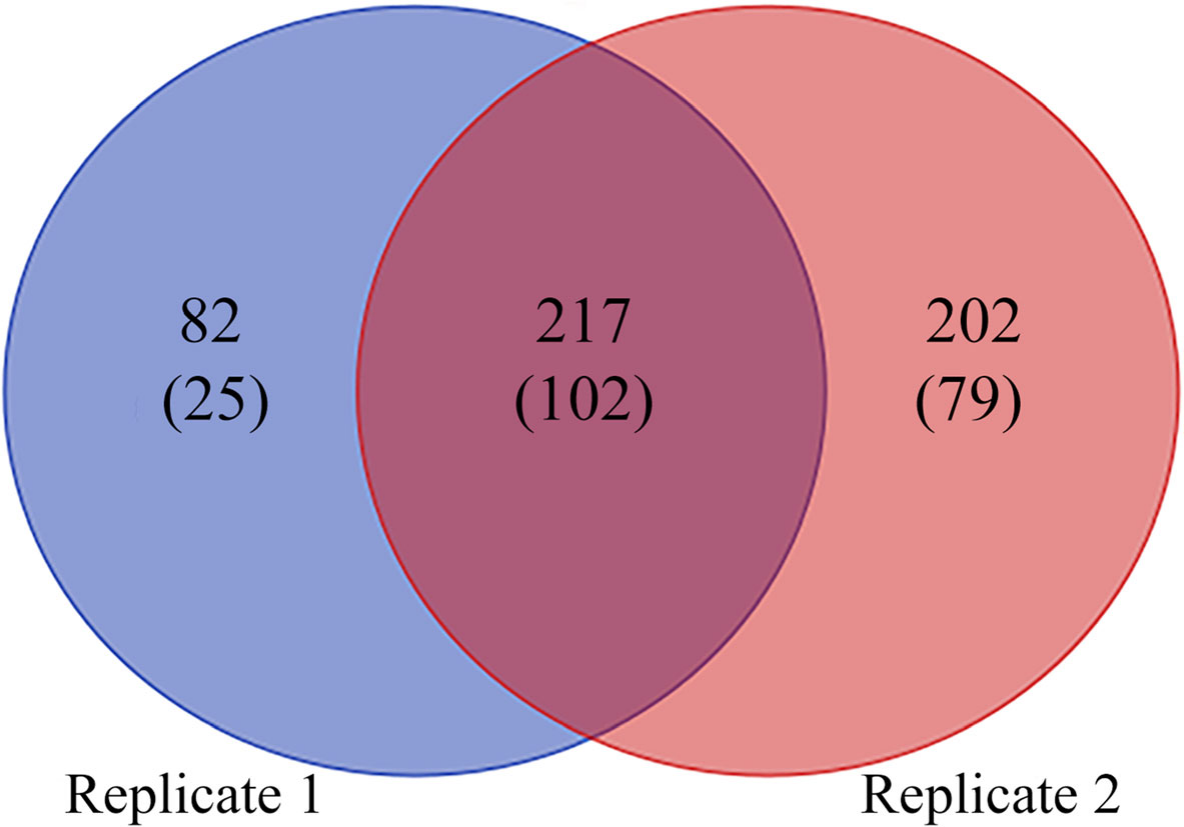
Venn diagram for the number of acetylation sites and proteins (in the bracket) in *Alvinocaris longirostris* from the two replicates

**Fig. 3:**
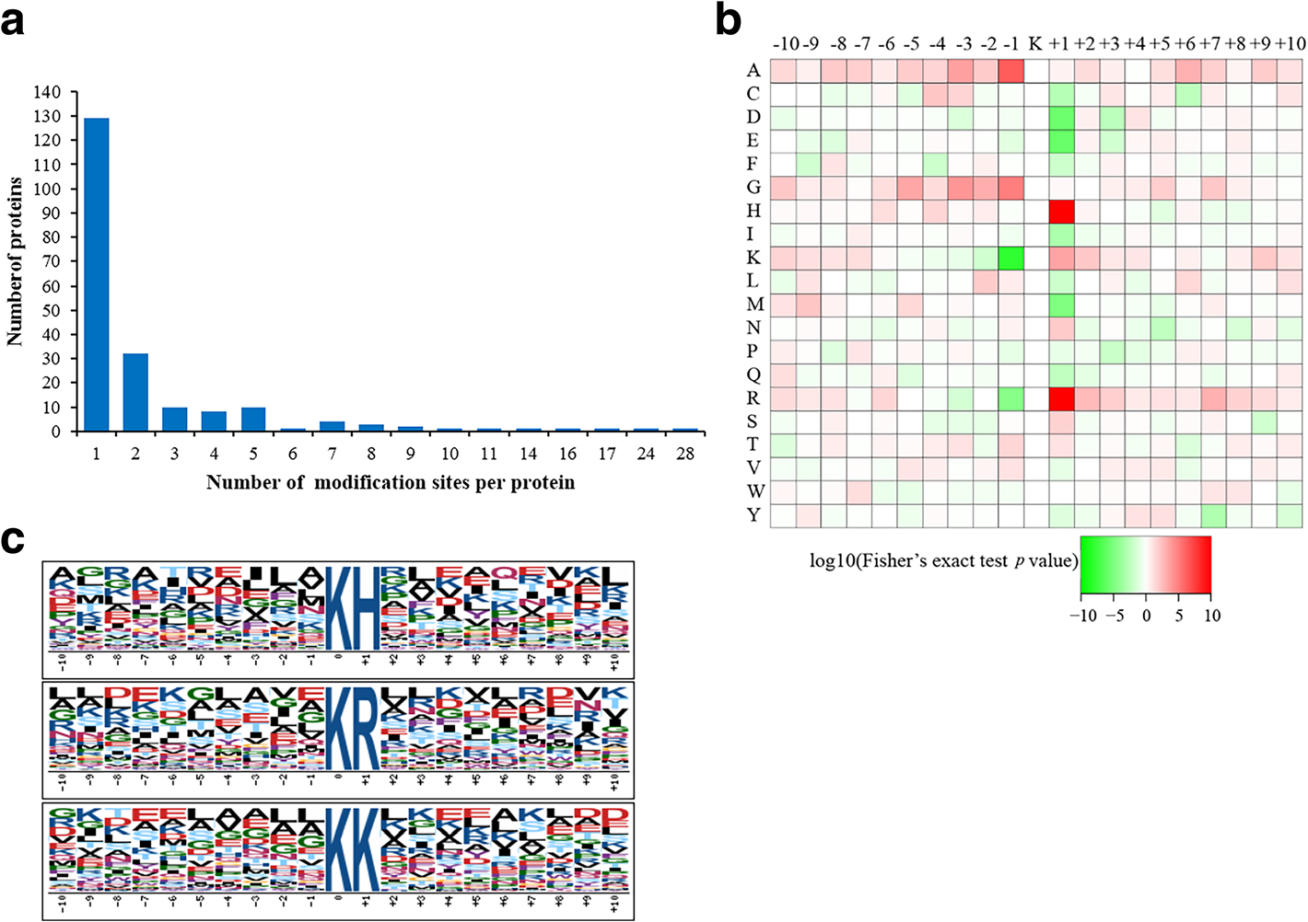
Characteristics of the lysine acetylation sites in *Alvinocaris longirostris*. **a** Distribution of the number of the lysine acetylation sites per protein. **b** Heat map of the amino acid compositions of the acetylation site motifs. The central K refers to the acetylated lysine. **c** Enriched acetylation motif logos. The size of each letter represents the frequency of the amino acid residue in that position

In the identification, the mass errors were lower than 5 ppm and the majorities were near zero (Fig. 1b), showing the mass accuracy of the MS data. The length of the peptides ranged from 7 to 34 amino acids and most were between 7 and 20 amino acids (Fig. 1c), which confirmed with the standard of sample preparation. Altogether 501 unique acetylation sites from 206 proteins were identified (Additional file 1: Table S1) with 217 sites and 102 proteins co-detected in the two experimental replicates (Fig. 2). All 501 acetylation sites were included in the following analyses. The numbers of modification sites per protein were from 1 to 28, in which more than 60% of proteins contained only one acetylation site (Fig. 3a), which was in common with many other studies [33, 36]. However, probably due to the limited genome information in *A. longirostris*, the numbers of detected acetylation sites and proteins are much less than those in *Drosophila* with well assembled whole genome sequences [33]. Additionally, only the abdomen has been selected for analysis in this study, and more tissues and organs should be included considering the tissue specific acetylation [11], which may contribute to the integrity of the acetylome. The acetylation level of proteins varying significantly between different species maybe another reasonable interpretation as suggested in previous studies [36, 37]. However, as the first lysine acetylome map of marine invertebrate and hydrothermal vent fauna, it is expected to supply valuable resources for PTM study in the future.

### Motif characters of the acetylated peptides in *A. longirostris*

To further explore the acetylation pattern in *A. longirostris*, flanking amino acid residues from position − 10 to + 10 around the acetylated lysine were analyzed. Strong bias in amino acids of specific acetylation site motifs was observed. Arg (KacR), His (KacH) and Lys (KacK) occurred most frequently at the + 1 position (Fig. 3b, c), which were all alkaline amino acids and positively charged. In the previous study for plants [20, 21] and bacteria [37, 38], KacH has been also identified in the consensus motif. However, these biases are different from those of *Drosophila* and human, in which no significant bias of specific motif is detected and the Tyr, Phe and Pro occur relatively more frequent at the + 1 position [33, 39]. Therefore, the motif analysis results suggest that acetylation preferentially occurs at alkaline and positively amino acid nearby regions in *A. longirostris*, which may be functionally important for acetylation in the shrimps.

### Functional annotation and enrichment of acetylated proteins in *A. longirostris*

**Fig. 4:**
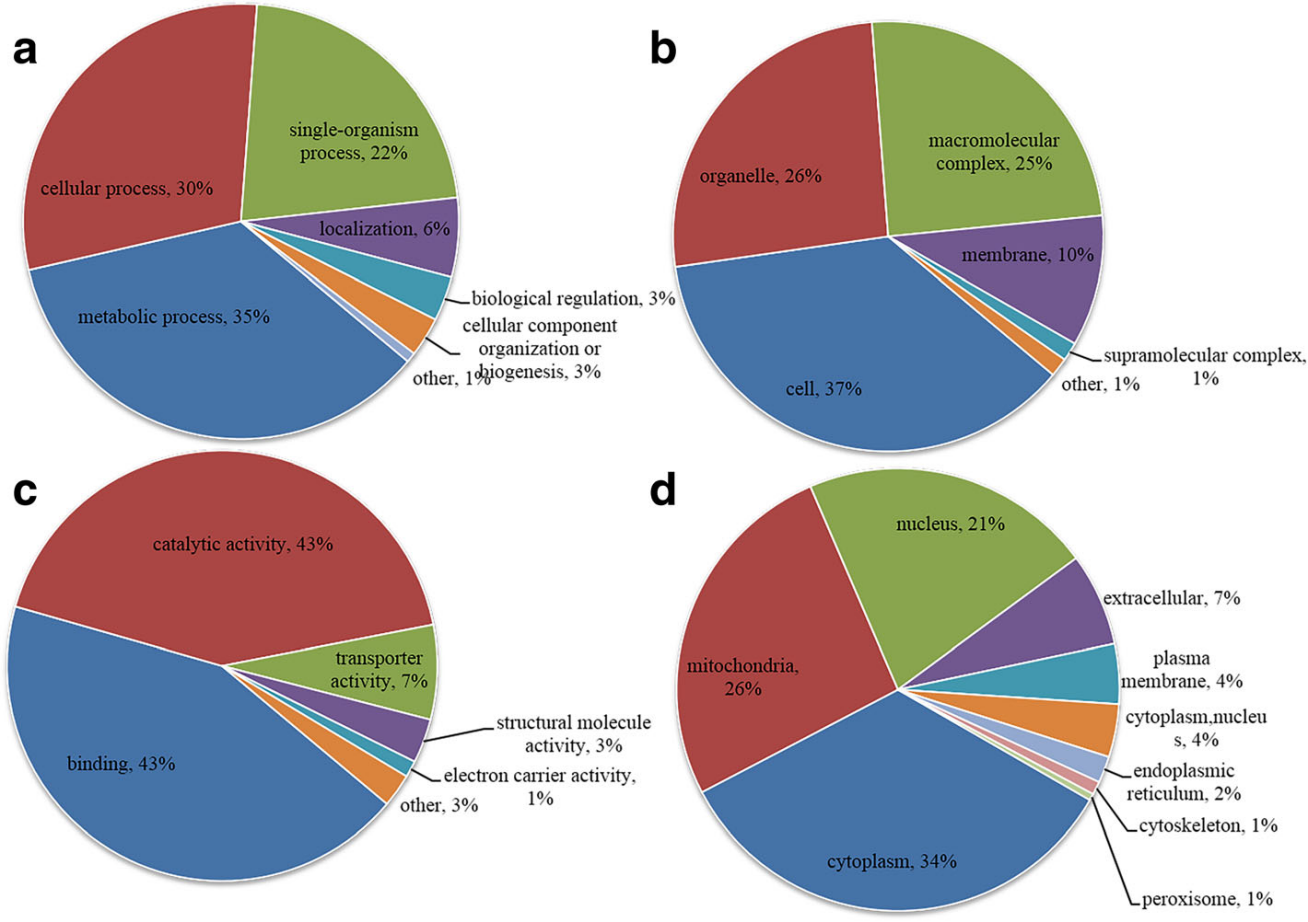
Functional distribution and subcellular localization of the lysine acetylated proteins in *Alvinocaris longirostris*. **a** Classification based on biological process. **b** Classification based on cellular component. **c** Classification based on molecular function. **d** Subcellular location prediction

GO analysis was performed to gain insight into the potential functional implications of acetylation in the shrimp. In total, 170 (82.52%) of the acetylated proteins were annotated and classified into three categories: ‘Biological process’, ‘Cellular component’ and ‘Molecular function’ (Fig. 4; Additional file 1: Table S1). In the ‘Biological process’ category, the most prevalent GO terms were metabolic process (35%), cellular process (30%) and single-organism process (22%), while localization, biological regulation, cellular component organization or biogenesis, and the other terms accounted for 6, 3, 3 and 1%, respectively (Fig. 4a). Cell (37%), organelle (26%) and macromolecular complex (25%) were the main ‘Cellular component’ (Fig. 4b). In the ‘Molecular function’ classification, we found that most acetylated proteins were related to catalytic activity (43%) and binding (43%) (Fig. 4c). According to the functional annotation, many metabolism related enzymes were identified to be acetylated (Additional file 1: Table S1), such as acyl-coenzyme A oxidase 3 (c94891_g1_orf1), citrate synthase (c77391_g1_orf1), glycogen phosphorylase (c75875_g1_orf1), fructose-bisphosphate aldolase (c73820_g1_orf1), malate dehydrogenase (c72173_g1_orf1, c90371_g1_orf1), phosphoglycerate kinase (c89458_g1_orf1), which possibly function in the cell metabolic regulation as previously revealed in various species [38, 40, 41]. In these enzymes, some of the homologous proteins in *Drosophila* and human have also been characterized to be lysine acetylated, indicating their evolutionary and functional conservation [33, 39].

**Fig. 5:**
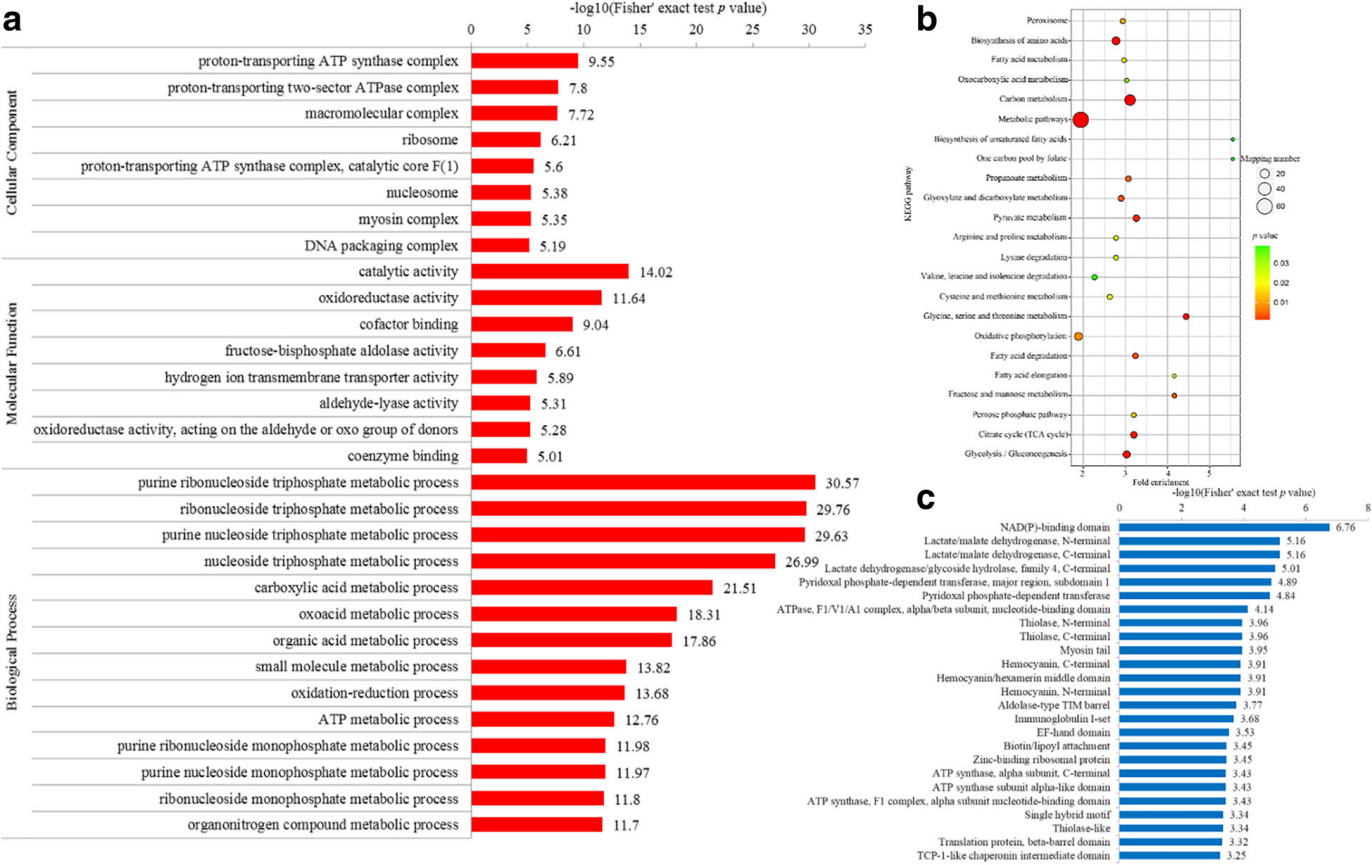
Enrichment analysis of the lysine acetylated proteins in *Alvinocaris longirostris*. **a** Enrichment based on GO annotation. **b** Enrichment based on KEGG pathways. **c** Enrichment based on protein domains

To further determine the proteins more prone to be acetylated, we enriched the acetylated proteins against all predicted proteins from the transcriptome. In the GO enrichment, it was noticed that several protein complexes were preferred to be acetylated, including the proton-transporting ATP synthase complexes (≥5.6%), macromolecular complex (7.72%), ribosome (6.21%), nucleosome (5.38), myosin complex (5.35%), and DNA packaging complex (5.19%) (Fig. 5a). Meanwhile, diverse molecular functions were enriched, such as catalytic activity (14.02%), oxidoreductase activity (11.64%), cofactor binding (9.04%) and fructose-bisphosphate aldolase activity (6.61%), which were involved in various metabolic processes (≥11.7%) and oxidation-reduction process (13.68%) (Fig. 5a). In the KEGG enrichment, 23 pathways were detected to be significantly enriched, e.g. carbon metabolism, biosynthesis of amino acids, citrate cycle, fatty acid degradation, oxidative phosphorylation and peroxisome (Fig. 5b). In the protein domains, thiolase, hemocyanin and TCP-1 like chaperonin intermediate domains were revealed to be significantly enriched, which might be involved in the adaptation to the high H_2_S and hypoxia, as well as toxin transport in the hydrothermal vents of *A. longirostris*. Therefore, lysine acetylation trends to target large macromolecular complexes as reported in human [39] and are associated with various process, like substance transport and metabolism, oxidation-reduction, protein synthesis, chromatin remodeling, which may function in the cell metabolism regulation and stress response to the hydrothermal vents of the shrimp. However, further experimental verification is indispensable. Since optimal conditions to assay unique sites deacetylation are formidable, pivotal acetylated proteins involved in certain biological process may be purified and selected for functional study by in vitro site-directed mutagenesis of lysine to arginine or glutamine initially [42–44].

### Abundant lysine acetylation in mitochondrion and peroxisome of *A. longirostris*

**Fig. 6:**
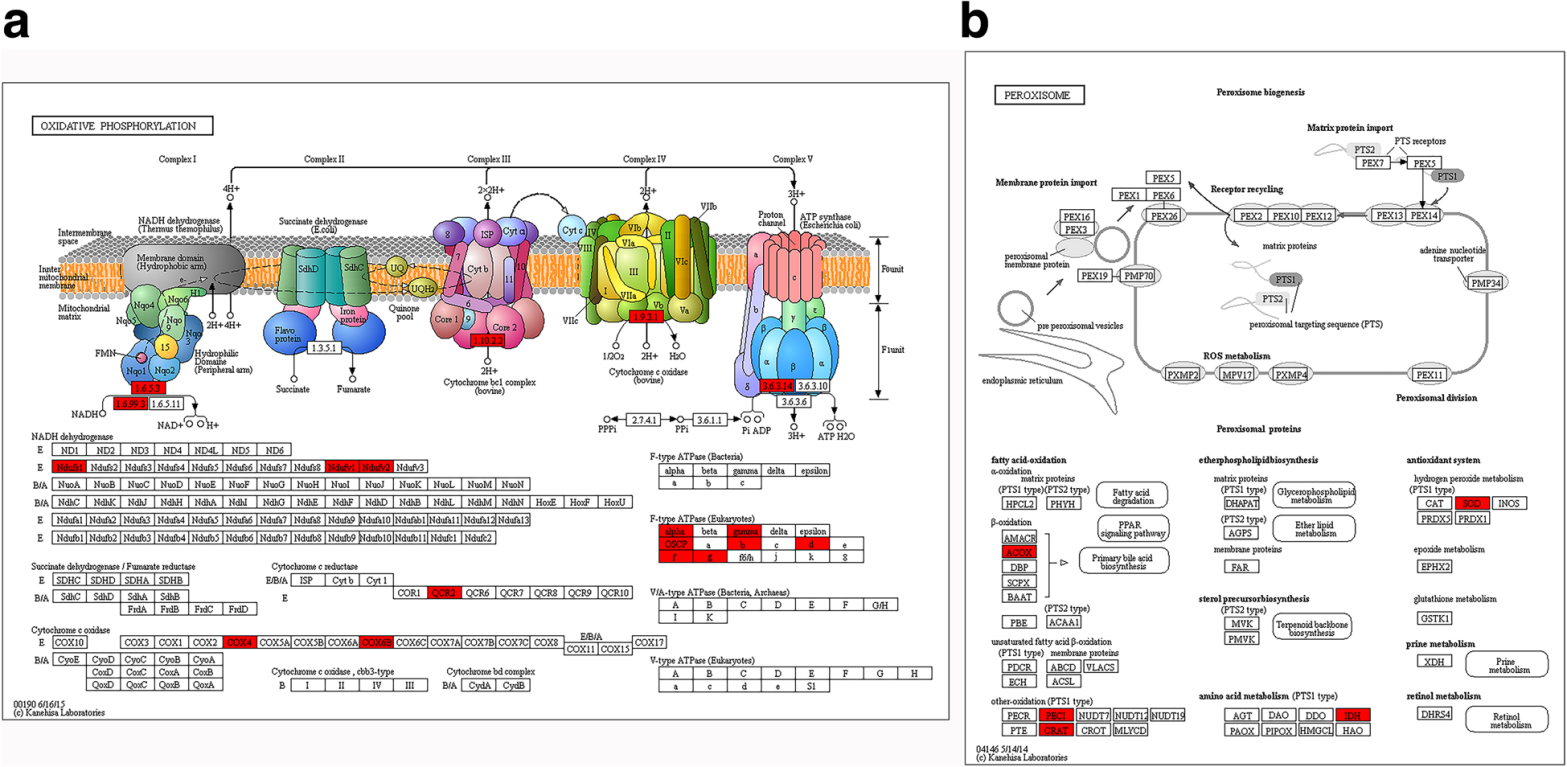
Significantly enriched KEGG pathways in mitochondrion and peroxisome. **a** Oxidative phosphorylation. **b** Peroxisome. The acetylated proteins are marked with red. The pictures are drawn by KEGG Mapper (www.kegg.jp/kegg/tool/map_pathway2.html)

Analysis of acetylated proteins subcellular localization showed that most of them were predicted to localize in cytoplasm (34%), mitochondria (26%) and nucleus (21%), while the others took a proportion of 19%. Previously, large-scale proteomics studies have demonstrated that lysine acetylation is widespread in mitochondria [10, 39, 45, 46], which is the key regulator of cellular energy production and pivotal in the regulation of metabolism and maintaining of cellular homeostasis. In this study, 14 acetylated proteins were identified to be involved in the oxidative phosphorylation which occurred in mitochondria (Fig. 6a), such as ATPase, Cytochrome c oxidase and reductase, and NADH dehydrogenase. Oxidative phosphorylation has been reported to play central roles in various stress response of many crustacean species by altering gene expression or protein activity [47–50]. Peroxisome is another ubiquitous subcellular organelle participating in metabolic and pathological/stress response processes [51, 52]. In the KEGG pathway enrichment, six acetylated proteins were discovered to be involved in peroxisome, which were acyl-CoA oxidase, carnitine O-acetyltransferase, carnitine O-octanoyltransferase, isocitrate dehydrogenase, superoxide dismutase, Fe-Mn family and peroxisomal 3,2-trans-enoyl-CoA isomerase (Fig. 6b). Limited PTMs have been identified in peroxisome thus far. Acetylation of a peroxisomal enzyme of peroxisomes has been explored in rat liver and suggests that acetylation may play a functional role in the organelle proliferative process [51]. We speculate that acetylation or deacetylation of these mitochondrial and peroxisome proteins may probably regulate the protein function in order to adapt to the hydrothermal vent environments in *A. longirostris*.

### Interaction network of acetylated proteins in *A. longirostris*

**Fig. 7:**
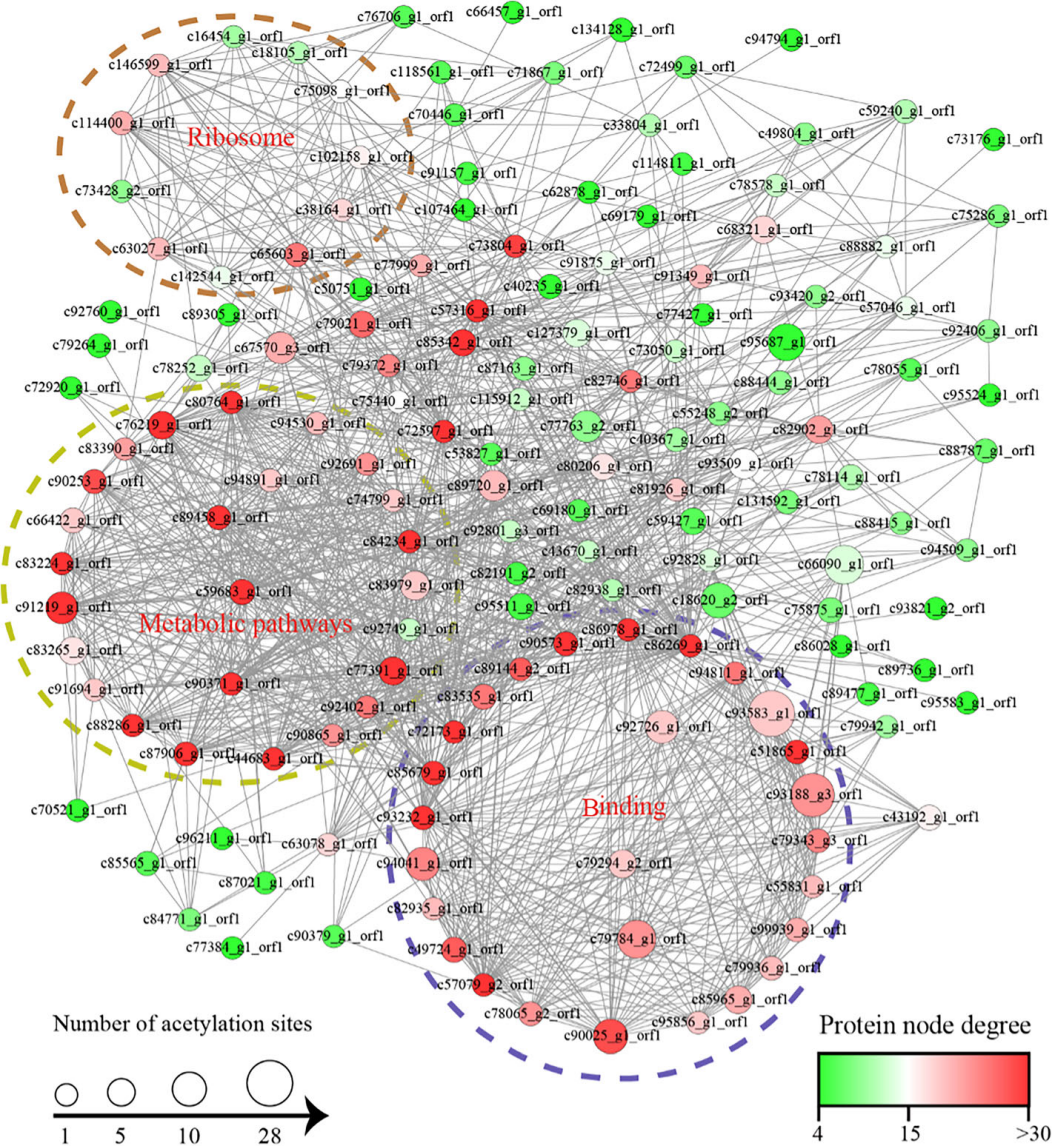
Protein-protein interaction network of all acetylated proteins in *Alvinocaris longirostris*

Protein interactions play an important part in various biological pathways and orchestrate virtually all cellular processes [53]. We further built the PPI network for all acetylated proteins in order to deeply investigate the processes regulated by acetylation and how these modified proteins mutually interwined. A total of 155 acetylated proteins were identified as nodes and connected with each other (Fig. 7, Additional file 2: Table S2), which represented a global PPI network of *A. longirostris*. The degree of node is the key parameter to evaluate the correlation of protein in network. Among them, six proteins displayed the highest degree (≥40, Additional file 2: Table S2), which were actin 6 (Act6), actin alpha cardiac muscle 1 (Actc1), DNA topoisomerase 2-beta (Top2b), delta-1-pyrroline-5-carboxylate dehydrogenase mitochondrial (Aldh4a1), 10-formyltetrahydrofolate dehydrogenase (Aldh1l1) and trifunctional enzyme subunit alpha mitochondrial (Hadha). By extraction of the highly enriched interaction clusters, we found that the top enriched Cluster 1 consisted proteins involved in binding function, while Cluster 2 and Cluster 3 were metabolism and ribosome associated proteins, respectively (Fig. 7, Additional file 2: Table S2). It was noted that Act6, Actc1 and Top2b were located in Cluster 1, whereas Aldh4a1, Aldh1l1 and Hadha were all included in metabolic pathways, Cluster 2. Massive lysine acetylation in different metabolic processes and ribosome also have been revealed from eukaryotes and prokaryotes [20, 54]. The potential acetylated protein interactions in this study provide valuable candidates in the following studies for their coordination and regulation in the hydrothermal vent shrimps.

### Acetylation associated with stress adaptation and hemocyanins in *A. longirostris*

Considering the challenging environment in hydrothermal vents where *A. longirostris* inhabit, such as low oxygen and high levels of toxic substances, we particularly analyzed the acetylated proteins which might be directly response to the stress. According to the annotation, two arginine kinases, Aks, (c18620_g2_orf1, c85882_g1_orf1), lethal (2) essential for life, L(2)efl (c40235_g1_orf1), heat shock protein 70, Hsp70 (c57316_g1_orf1) and hemocyanins (c93924_g1_orf1, c95510_g4_orf1) were identified (Additional file 1: Table S1). As phosphagen kinase, Aks mainly distribute in invertebrates, and play critical roles in the energy mechanism, as well as various stress responses [55]. In *Macrobrachium rosenbergii*, Ak has been revealed involved in the immune response of this shrimp [56]. In *A. longirostris*, Ak1 and Ak2 displayed hyper-acetylation with 11 and 5 acetylation sites, respectively, which may contribute to the physiological regulation in the adaptation to the extreme environments. Moreover, L(2)efl and Hsp70 with acetylation sites both belong to HSP family, which were well-known for their function in cell response to stress, such as thermal stimulus and osmotic pressure [48, 57, 58].

**Fig. 8:**
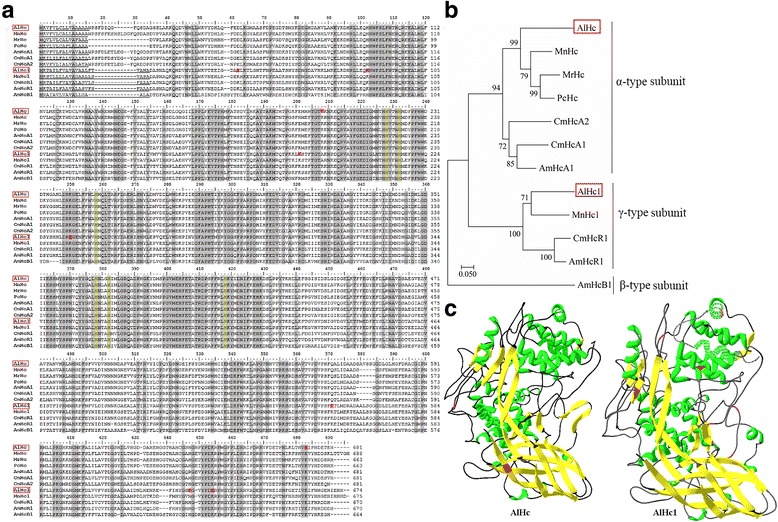
Sequence alignment and phylogenetic tree of hemocyanins from selected caridea species. **a** Multiple sequence alignment. The sequences are from *Alvinocaris longirostris* (AlHc, c93924_g1_orf1; AlHc1, c95510_g4_orf1), *Atyopsis moluccensis* (AmHcA1, CCF55379.1; AmHcB1, CCF55382.1; AmHcR1, CCF55383.1), *Caridina multidentata* (CmHcA1, CCF55384.1; CmHcA2, CCF55385.1; CmHcR1, CCF55387.1), *Macrobrachium nipponense* (MnHc, AHJ90473.1; MnHc1, AGA17871.1), *M. rosenbergii* (MrHc, ALN67306.1), and *Palaemon carinicauda* (PcHc, AEJ08191.1). Strictly conserved regions are shaded grey and the conserved copper-binding histidines are marked with yellow. Potential signal regions are underlined. The acetylation sites in hemocyanins of *A. longirostris* are labeled with red. **b** Phylogenetic tree of the hemocyanins. **c** Predicted three-dimensional structure of hemocyanins in *A. longirostris*. The acetylation sites are marked with red

Oxygen (O_2_) transport is an essential biological process in animals. In the deep-sea hydrothermal vent environments, there are lack of O_2_ and therefore specific regulation mechanism in organisms is compulsory in order to adapt to the hypoxia. Hemocyanin (Hc) is one of the key respiratory proteins in the blood of arthropods [59], with a principal structure of a hexamer of six similar subunits [60]. Recent studies show that Hc potentially also involved in other stress response of decapods [6, 61–63]. In another alvinocaridid shrimp, *Rimicaris exoculata*, Hc encoding gene shows a strong up-regulation when the shrimp is exposed to heat stress [64]. Evidence of acetylation in Hcs has been suggested previously [65, 66], but the information is quite limited. In this study, two Hcs were revealed to be acetylated, named AlHc and AlHc1 with two and seven acetylation sites, respectively. The deduced amino acids (aa) of AlHc and AlHc1 contained 681 and 674 aa (Fig. 8a). Two Hc sequences in *A. longirostris* were then aligned with 10 Hc sequences of other Caridea species. They all contained six conserved copper-binding histidines and a signal peptide (Fig. 8a). Hc sequences of *A. longirostris* showed the highest similarity with those of *M. nipponense*, which were also supported by the phylogenetic tree (Fig. 8b).

Previously, three distinct types of hemocyanin subunits have been defined and referred to α, β and γ, in which α and γ are more related with each other than with β [67, 68]. It was obvious that AlHc and AlHc1 were classified into α and γ types based on the tree (Fig. 8b). By second structure analysis, we found site 199 with acetylation of AlHc was located in coli, while site 673 with acetylation was in β-strand (Fig. 8c). In AlHc1, two acetylation sites (sites 47, 555) were contained in α-helix, one in β-strand (site 185), and four (sites 87, 234, 628, 634) in coli (Fig. 8c). It indicates that acetylation in Hcs of *A. longirostris* prefers to occur at regions with ordered secondary structure, which may be involved in the stability of the Hc complex. On the other hand, more acetylation sites were discovered in AlHc1 than those in AlHc of *A. longirostris*. Moreover, the acetylation site 628 in AlHc1 was unique in *A. longirostris* when compared with other shallow water shrimps (Fig. 8a), K (acetylated) in *A. longirostris* whereas H, N, or R in other species. We infer that this lysine acetylation probably participate in the specific adaptation to the hydrothermal vent environments in *A. longirostris*, but further verification is essential.

### Acetylation of microbes associated with abdomen of *A. longirostris*

At the base of the food web, microbes are thriving in the extreme hydrothermal vent and cold seep environments, which are dominated by chemoautotrophic bacteria and archaea fuelled by simple reduced molecules [69]. The symbiont between invertebrates and microbes is also critical for the survival of marcoorganisms. Therefore, it is interesting to investigate PTMs in these microbes and their potential roles in the adaptation to the extreme conditions and in the symbiont with the host. However, we have no protein database of microbes from the *A. longirostris* in Iheya North hydrothermal vent area. Methylococcaceae has been discovered rich in a deep-sea mussel *Bathymodiolus platifrons*, another species co-distributed in hydrothermal vent and methane seep [4]. Considering the methane concentration is also high in our sampling site, the mass spectra have been only searched against the protein database of the family Methylococcaceae in order to narrow the range.

Totally, 32 unique acetylation sites from 23 proteins were identified (Additional file 3: Table S3), including methanol dehydrogenase associated peptide, ABC transporter permease, Trk system potassium uptake protein, peptidase M16, ATP-dependent RNA helicase, translocase subunit, 3′,5′-cyclic AMP phosphodiesterase, DNA polymerase I, IS256 family transposase, ATPase of the AAA^+^ class, tyrosine recombinase, chromosome partition protein and other uncharacterized proteins. According to the GO annotation, these PTMs are probably participate in the adaptation to the extreme environment of microbes mainly by regulating methane oxidation, cell redox homeostasis, substance transmembrane transport and DNA repair. It is essential to obtain the exact protein and acetylation data of microbes from different tissues of the host, such as gill, seta and gut, where more microbes are available. However, we have firstly identified candidate acetylation sites in the microbes of deep-sea chemosynthetic ecosystems preliminarily. Intensive study on the PTMs in the microbes is also expected to facilitate clarifying the symbiont mechanism.

## Conclusion

In conclusion, this work provides the first global lysine acetylation data in the deep-sea hydrothermal vent shrimp, *A. longirostris* and even in the marine invertebrates. A total of 501 unique acetylation sites from 206 proteins were identified. Three consensus sequence motifs were extracted. Functional analysis revealed that the protein acetylation was involved in diverse metabolic pathways, oxidative phosphorylation, and many other cellular processes. Abundant lysine acetylation was discovered in mitochondrion and peroxisome. Acetylation associated with stress response was further detected and the acetylation of key respiratory protein Hcs was specifically characterized. Therefore, our study provides new insights into the regulation function of the reversible lysine acetylation, and supplies rich candidates for exploring the functions of acetylation in the adaptation to hydrothermal vents in *A. longirostris*, and in other shrimps.

## Additional files

Additional file 1: **Table S1.** Information for the acetylated proteins in *Alvinocaris longirostris*. (XLSX 143 kb)

Additional file 2: **Table S2.** Information for the protein-protein interaction network in *Alvinocaris longirostris*. (XLSX 20 kb)

Additional file 3: **Table S3.** Information for the acetylation proteins of microbes associated with abdomen of *Alvinocaris longirostris*. (XLSX 15 kb)

## Abbreviations

Act6: Actin 6
Actc1: Actin alpha cardiac muscle 1
AGC: Automatic gain control
Aks: Arginine kinases
Aldh1l1: 10-formyltetrahydrofolate dehydrogenase
Aldh4a1: Delta-1-pyrroline-5-carboxylate dehydrogenase mitochondrial
AlHc: *Alvinocaris longirostris* hemocyanin
AlHc1: *Alvinocaris longirostris* hemocyanin subunit 1
CH_4_: Methane
FDR: False discovery rate
GO: Gene Ontology
H_2_S: Hydrogen sulfide
Hadha: Trifunctional enzyme subunit alpha mitochondrial
Hc: Hemocyanin
Hsp70: Heat shock protein 70
IACUC: Institutional Animal Care and Use Committee
KEGG: Kyoto Encyclopedia of Genes and Genomes
L(2)efl: Lethal (2) essential for life
LC: Liquid chromatography
MCODE: Molecular complex detection
MS: Mass spectrum
NCE: Normalized collisional energy
NSI: Nanospray ionization
O_2_: Oxygen
PPI: Protein-protein interaction
PTMs: Post-translational modifications
ROV: Remotely operated vehicle
RV: Research vessel
Top2b: DNA topoisomerase 2-beta

## References

[1] Little CTS, Vrijenhoek RC (2003). Are hydrothermal vent animals living fossils?. Trends Ecol Evol.

[2] Bettencourt R, Pinheiro M, Egas C, Gomes P, Afonso M, Shank T, Santos RS (2010). High-throughput sequencing and analysis of the gill tissue transcriptome from the deep-sea hydrothermal vent mussel *Bathymodiolus azoricus*. BMC Genomics.

[3] Zheng P, Wang M, Li C, Sun X, Wang X, Sun Y, Sun S (2017). Insights into deep-sea adaptations and host-symbiont interactions: a comparative transcriptome study on *Bathymodiolus* mussels and their coastal relatives. Mol Ecol.

[4] Sun J, Zhang Y, Xu T, Zhang Y, Mu H, Zhang Y, Lan Y, Fields CJ, Hui J, Zhang W (2017). Adaptation to deep-sea chemosynthetic environments as revealed by mussel genomes. Nat Ecol Evol.

[5] Zhang Y, Sun J, Chen C, Watanabe HK, Feng D, Zhang Y, Chiu JMY, Qian PY, Qiu JW (2017). Adaptation and evolution of deep-sea scale worms (Annelida: Polynoidae): insights from transcriptome comparison with a shallow-water species. Sci Rep.

[6] Hui M, Song C, Liu Y, Li C, Cui Z (2017). Exploring the molecular basis of adaptive evolution in hydrothermal vent crab *Austinograea alayseae* by transcriptome analysis. PLoS One.

[7] Zhang J, Sun QL, Luan ZD, Lian C, Sun L (2017). Comparative transcriptome analysis of *Rimicaris* sp. reveals novel molecular features associated with survival in deep-sea hydrothermal vent. Sci Rep.

[8] Mann M, Jensen ON (2003). Proteomic analysis of post-translational modifications. Nat Biotechnol.

[9] Karve TM, Cheema AK (2011). Small changes huge impact: the role of protein posttranslational modifications in cellular homeostasis and disease. J Amino Acids.

[10] Kim SC, Sprung R, Chen Y, Xu Y, Ball H, Pei J, Cheng T, Kho Y, Xiao H, Xiao L (2006). Substrate and functional diversity of lysine acetylation revealed by a proteomics survey. Mol Cell.

[11] Lundby A, Lage K, Weinert BT, Bekkerjensen DB, Secher A, Skovgaard T, Kelstrup CD, Dmytriyev A, Choudhary C, Lundby C (2012). Proteomic analysis of lysine acetylation sites in rat tissues revealsorgan specificity and subcellular patterns. Cell Rep.

[12] Rodgers JT, Lerin C, Gerhart-Hines Z, Puigserver P (2008). Metabolic adaptations through the PGC-1α and SIRT1 pathways. FEBS Lett.

[13] Wang Q, Zhang Y, Yang C, Lin Y, Yao J, Li H, Xie L, Zhao W, Yao Y, Ning ZB (2010). Acetylation of metabolic enzymes coordinates carbon source utilization and metabolic flux. Science.

[14] Schwer B, Bunkenborg J, Verdin RO, Andersen JS, Verdin E. Reversible lysine acetylation controls the activity of the mitochondrial enzyme acetyl-coa synthetase 2. Proc Natl Acad Sci U S A. 2006;103:10224–9. 10.1073/pnas.0603968103 PMC1502439 16788062

[15] Lee CF, Tian R (2015). Mitochondrion as a target for heart failure therapy-role of protein lysine acetylation. Circ J.

[16] Hosp F, Lassowskat I, Santoro V, De VD, Fliegner D, Redestig H, Mann M, Christian S, Hannah MA, Finkemeier I (2017). Lysine acetylation in mitochondria: from inventory to function. Mitochondrion.

[17] Sol EM, Wagner SA, Weinert BT, Kumar A, Kim HS, Deng CX, Choudhary C (2012). Proteomic investigations of lysine acetylation identify diverse substrates of mitochondrial deacetylase Sirt3. PLoS One.

[18] Shyama Prasad Rao R, Thelen JJ, Miernyk JA (2014). *In silico* analysis of protein Lys-N^Ɛ^-acetylation in plants. Front Plant Sci.

[19] Shyama Prasad Rao R, Thelen JJ, Miernyk JA (2014). Is Lys-N^ɛ^-acetylation the next big thing in post-translational modifications?. Trends Plant Sci.

[20] Fang X, Chen W, Zhao Y, Ruan S, Zhang H, Yan C, Jin L, Cao L, Zhu J, Ma H (2015). Global analysis of lysine acetylation in strawberry leaves. Front Plant Sci.

[21] Zhang Y, Song L, Liang W, Mu P, Wang S, Lin Q (2016). Comprehensive profiling of lysine acetylproteome analysis reveals diverse functions of lysine acetylation in common wheat. Sci Rep.

[22] Martin JW, Haney TA (2010). Decapod crustaceans from hydrothermal vents and cold seeps: a review through 2005. Zool J Linnean Soc.

[23] Tokuda G, Yamada A, Nakano K, Arita N, Yamasaki H (2006). Occurrence and recent long-distance dispersal of deep-sea hydrothermal vent shrimps. Biol Lett.

[24] Komai T, Segonzac M (2015). Taxonomic review of the hydrothermal vent shrimp Genera *Rimicaris* Williams & Rona and *Chorocaris* Martin & Hessler (Crustacea: Decapoda: Caridea: Alvinocarididae). J Shellfish Res.

[25] Watabe H, Miyake H (2000). Decapod fauna of the hydrothermally active and adjacent fields on the Hatoma Knoll, southern Japan. JAMSTEC J Deep Sea Res.

[26] Cox J, Mann M (2008). MaxQuant enables high peptide identification rates, individualized p.p.b.-range mass accuracies and proteome-wide protein quantification. Nat Biotechnol.

[27] Cox J, Matic I, Hilger M, Nagaraj N, Selbach M, Olsen JV, Mann M (2009). A practical guide to the MaxQuant computational platform for SILAC-based quantitative proteomics. Nat Protoc.

[28] Horton P, Park KJ, Obayashi T, Fujita N, Harada H, Adamscollier CJ, Nakai K (2007). WoLF PSORT: protein localization predictor. Nucleic Acids Res.

[29] Shannon P, Markiel A, Ozier O, Baliga NS, Wang JT, Ramage D, Amin N, Schwikowski B, Ideker T (2003). Cytoscape: a software environment for integrated models of biomolecular interaction networks. Genome Res.

[30] Morten N, Pernille A, Thomas P, Bent P, Claus L (2009). A generic method for assignment of reliability scores applied to solvent accessibility predictions. BMC Struct Biol.

[31] Tamura K, Stecher G, Peterson D, Filipski A, Kumar S (2013). MEGA6: molecular evolutionary genetics analysis version 6.0. Mol Biol Evol.

[32] Stamatakis A, Hoover P, Rougemont J, Renner S (2008). A rapid bootstrap algorithm for the RAxML web servers. Syst Biol.

[33] Weinert BT, Wagner SA, Horn H, Henriksen P, Liu WR, Olsen JV, Jensen LJ, Choudhary C (2011). Proteome-wide mapping of the *Drosophila* acetylome demonstrates a high degree of conservation of lysine acetylation. Sci Signal.

[34] Bharathi SS, Zhang Y, Mohsen AW, Uppala R, Balasubramani M, Schreiber E, Uechi G, Beck ME, Rardin MJ, Vockley J (2013). Sirtuin 3 (SIRT3) protein regulates long-chain acyl-CoA dehydrogenase by deacetylating conserved lysines near the active site. J Biol Chem.

[35] Still AJ, Floyd BJ, Hebert AS, Bingman CA, Carson JJ, Gunderson DR, Dolan BK, Grimsrud PA, Dittenhaferreed KE, Stapleton DS (2013). Quantification of mitochondrial acetylation dynamics highlights prominent sites of metabolic regulation. J Biol Chem.

[36] Zhou S, Yang Q, Yin C, Liu L, Liang W (2016). Systematic analysis of the lysine acetylome in *Fusarium graminearum*. BMC Genomics.

[37] Pan J, Ye Z, Cheng Z, Peng X, Wen L, Zhao F (2014). Systematic analysis of the lysine acetylome in *Vibrio parahemolyticus*. J Proteome Res.

[38] Zhang J, Sprung R, Pei J, Tan X, Kim S, Zhu H, Liu CF, Grishin NV, Zhao Y (2009). Lysine acetylation is a highly abundant and evolutionarily conserved modification in *Escherichia coli*. Mol Cell Proteomics.

[39] Choudhary C, Kumar C, Gnad F, Nielsen ML, Rehman M, Walther TC, Olsen JV, Mann M (2009). Lysine acetylation targets protein complexes and co-regulates major cellular functions. Science.

[40] Wang Q, Zhang Y, Yang C, Xiong H, Lin Y, Yao J, Li H, Xie L, Zhao W, Yao Y (2010). Acetylation of metabolic enzymes coordinates carbon source utilization and metabolic flux. Science.

[41] Zhao S, Xu W, Jiang W, Yu W, Lin Y, Zhang T, Yao J, Zhou L, Zeng Y, Li H (2010). Regulation of cellular metabolism by protein lysine acetylation. Science.

[42] Song L, Wang G, Malhotra A, Deutscher MP, Liang W (2016). Reversible acetylation on Lys501 regulates the activity of RNase II. Nucleic Acids Res.

[43] Ye X, Niu X, Gu L, Xu Y, Li Z, Yu Y, Chen Z, Lu S (2017). Desuccinylation of pyruvate kinase M2 by SIRT5 contributes to antioxidant response and tumor growth. Oncotarget.

[44] Zhao D, Zou SW, Liu Y, Zhou X, Mo Y, Wang P, Xu YH, Dong B, Xiong Y, Lei QY, Guan KL (2013). Lysine-5 acetylation negatively regulates lactate dehydrogenase A and is decreased in pancreatic cancer. Cancer Cell.

[45] Rardin MJ, Newman JC, Held JM, Cusack MP, Sorensen DJ, Li B, Schilling B, Mooney SD, Kahn CR, Verdin E (2013). Label-free quantitative proteomics of the lysine acetylome in mitochondria identifies substrates of SIRT3 in metabolic pathways. Proc Natl Acad Sci U S A.

[46] Gibson BW (2005). The human mitochondrial proteome: oxidative stress, protein modifications and oxidative phosphorylation. Int J Biochem Cell Biol.

[47] Morris S, van Aardt WJ, Ahern MD (2005). The effect of lead on the metabolic and energetic status of the Yabby, *Cherax destructor*, during environmental hypoxia. Aquat Toxicol.

[48] Hui M, Liu Y, Song C, Li Y, Shi G, Cui Z (2014). Transcriptome changes in *Eriocheir sinensis* megalopae after desalination provide insights into osmoregulation and stress adaption in larvae. PLoS One.

[49] Silvestre F, Trausch G, Devos P (2005). Hyper-osmoregulatory capacity of the Chinese mitten crab (*Eriocheir sinensis*) exposed to cadmium; acclimation during chronic exposure. Comp Biochem Phys C.

[50] Li TD, Brouwer M (2009). Gene expression profile of grass shrimp Palaemonetes pugio exposed to chronic hypoxia. Comp Biochem Phys D.

[51] Contreras MA, Alzate O, Singh AK, Singh I (2014). PPARα activation induces *N*^Ɛ^-Lys-Acetylation of rat liver peroxisomal multifunctional enzyme type 1. Lipids.

[52] Ricote M, Li AC, Willson TM, Kelly CJ, Glass CK (1998). The peroxisome proliferator-activated receptor-gamma is a negative regulator of macrophage activation. Nature.

[53] Wang H, Huang H, Ding C, Nie F (2013). Predicting protein-protein interactions from multimodal biological data sources via nonnegative matrix tri-factorization. J Comput Biol.

[54] Zhang K, Zheng S, Yang JS, Chen Y, Cheng Z (2013). Comprehensive profiling of protein lysine acetylation in *Escherichia coli*. J Proteome Res.

[55] Shofer SL, Willis JA, Tjeerdema RS (1997). Effects of hypoxia and toxicant exposure on arginine kinase function as measured by 31 P-NMR magnetization transfer in living abalone. Comp Biochem Phys C.

[56] Arockiaraj J, Vanaraja P, Easwvaran S, Singh A, Alinejaid T, Othman RY, Bhassu S (2011). Gene profiling and characterization of arginine kinase-1 (MrAK-1) from freshwater giant prawn (*Macrobrachium rosenbergii*). Fish Shellfish Immun.

[57] Zhang Y, Sun J, Mu H, Li J, Zhang Y, Xu F, Xiang Z, Qian PY, Qiu JW, Yu Z (2015). Proteomic basis of stress responses in the gills of the pacific oyster *Crassostrea gigas*. J Proteome Res.

[58] Kurzik-Dumke U, Lohmann E (1995). Sequence of the new *Drosophila melanogaster* small heat-shock-related gene, *lethal(2) essential for life* [*l(2)efl*], at locus 59F4,5. Gene.

[59] Magnus KA, Tonthat H, Carpenter JE (1994). Recent structural work on the oxygen transport protein hemocyanin. Chem Rev.

[60] Burmester T (2001). Molecular evolution of the arthropod hemocyanin superfamily. Mol Biol Evol.

[61] Zhang YL, Fang Y, Zhong H, Zhao XL, Min SY, Du ZH, Shan Z, Ye XQ, Li YY (2009). Hemocyanin from shrimp *Litopenaeus vannamei* shows hemolytic activity. Fish Shellfish Immun.

[62] Nagai T, Osaki T, Kawabata S (2001). Functional conversion of hemocyanin to phenoloxidase by horseshoe crab antimicrobial peptides. J Biol Chem.

[63] Sun S, Chen L, Qin J, Ye J, Qin C, Jiang H, Li E (2012). Molecular cloning, characterization and mRNA expression of copper-binding protein hemocyanin subunit in Chinese mitten crab, *Eriocheir sinensis*. Fish Shellfish Immun.

[64] Cottin D, Shillito B, Chertemps T, Tanguy A, Léger N, Ravaux J (2010). Identification of differentially expressed genes in the hydrothermal vent shrimp *Rimicaris exoculata* exposed to heat stress. Mar Genomics.

[65] Zatta P, Salvato B (1981). Evidence for acetylated amino-terminal residues in hemocyanins. Boll Soc Ital Biol Sper.

[66] Herskovits TT, Carberry SE, George RCS (1983). Subunit structure and dissociation of *Homarus americanus* hemocyanin: effects of salts and ureas on the acetylated and unmodified hexamers. Biochemistry.

[67] Markl J (1986). Evolution and function of structurally diverse subunits in the respiratory protein hemocyanin from arthropods. Biol Bull.

[68] Markl J, Stöcker W, Runzler R, Precht E, Linzen B (1986). Immunological correspondences between the hemocyanin subunits of 86 arthropods: evolution of a multigene protein family. Invertebrate oxygen carriers.

[69] Huber JA, Mark Welch DB, Morrison HG, Huse SM, Neal PR, Butterfield DA, Sogin ML (2007). Microbial population structures in the deep marine biosphere. Science.

